# Has the United States Reached a Plateau in Overdoses Caused by Synthetic Opioids After the Onset of the COVID-19 Pandemic? Examination of Centers for Disease Control and Prevention Data to November 2021

**DOI:** 10.3389/fpsyt.2022.947603

**Published:** 2022-07-07

**Authors:** Kate G. Brown, Carina Y. Chen, Deanna Dong, Kimberly J. Lake, Eduardo R. Butelman

**Affiliations:** Laboratory on the Biology of Addictive Diseases, The Rockefeller University, New York, NY, United States

**Keywords:** COVID-19, overdose, opioid use disorder, fentanyl, plateau, opioid

## Abstract

**Background:**

Overdoses caused by synthetic mu-opioid receptor (MOR) agonists such as fentanyl are causing increasing mortality in the United States. The COVID-19 pandemic continues to have complex effects on public health, including opioid use disorders (OUD). It is unclear whether recent increases in mortality caused by synthetic opioids have reached a plateau (i.e., a stable period), after the onset of the COVID-19 pandemic.

**Method:**

This study examined provisional overdose mortality data from the Centers for Disease Control and Prevention, for synthetic opioids excluding methadone (code T40.4; monthly data available from 39 States, plus New York City and Washington DC), for June 2019–November 2021. Data were first examined as crude mortality rates. The presence of a maximum plateau was analyzed for the last 4 months of available data. For authorities in which a plateau in mortality was detected, sigmoidal Boltzmann equations were used to model parameters of this phenomenon (e.g., level of the plateau).

**Results:**

At the end of the study period, all but one authority (New Hampshire) reported increases in mortality rates for synthetic opioids, compared to the baseline month of June 2019 (range: 111–745% of baseline). A plateau was observed over the last 4 months of the study period (Aug 2021–Nov 2021) in 29 of the authorities. Ten other authorities had not reached a stable plateau at the end of the study period. For the authorities where a plateau was detected, a sigmoidal Boltzmann model revealed a fitted maximum of 262% rise in mortality over the study period, from the baseline month. The midpoint in the rise in mortality was fitted in September 2020. After separation of data into census regions, the highest plateau was observed in the West region, followed by South, Midwest, and Northeast (fitted plateau values were 409, 262, 204, and 149% of baseline, respectively).

**Discussion:**

There were increases in overdose mortality due to synthetic opioids across most states, ranging considerably in magnitude. A plateau in overdose mortality was detected at the end of the study period in most of these authorities. The reasons for these plateaus should be explored, in order to develop optimized public health interventions.

## Introduction

Starting with its global spread from early 2020, the COVID-19 pandemic has caused complex public health challenges. Most public health measures (e.g., lockdowns) in industrialized countries such as the United States commenced in March 2020 (1). Among the reported comorbidities with COVID-19 is opioid use disorder (OUD) (2). The COVID-19 pandemic could potentially affect different stages in the trajectory of OUD and its medical care (3–5). OUD is caused by self-exposure to mu-opioid receptor (MOR) agonists, such as fentanyl derivatives, heroin, and prescription opioids (e.g., oxycodone). MOR-agonist overdose causes mortality primarily by centrally mediated respiratory depression (6–9). Fentanyl analogs have other deleterious effects (e.g., chest rigidity and laryngospasm), which may further increase their potential to cause morbidity and mortality (10–13).

Even prior to the COVID-19 pandemic, increases in opioid-induced overdose mortality were reported (14–16). For example, a recent wave in 2014–2017 was thought to be due to fentanyl derivatives, heroin and prescription opioids (17). The increasing presence of synthetic opioids in the illicit drug supply has resulted in a substantial increase in overdoses and mortality in the United States over the last several years (18–22). Consistent with this, data indicate that synthetic opioids (e.g., fentanyl derivatives), as opposed to other MOR-agonists such as heroin, are primarily responsible for the most recent increases in mortality in the United States, after the onset of COVID-19 (14, 23, 24).

The time-dependent increase in opioid-induced overdoses after the onset of COVID-19 was recently examined in 11 states (ending in August–December 2020) (25). This study reported heterogeneous profiles across these 11 states, for different kinds of opioids, including potential stabilizing trends in some states (i.e., tending toward a plateau). Another study reported on the increases in suspected opioid overdoses visits to ED in four states after onset of the COVID-19 lockdowns, ending in August 2020 (5). Other studies have also reported increases in all drug overdoses in individual states and counties in the onset of the COVID-19 pandemic (26–29).

The present study extends and complements the above reports by examining U.S. Centers for Disease Control (CDC) data from a larger national sample (39 states, plus New York City and Washington DC), on mortality due to synthetic opioids, over a more prolonged period after the onset of COVID-19 (starting June 2019 and ending November 2021). Furthermore, this study directly focused on whether plateaus in mortality have occurred across states (i.e., whether mortality levels have stabilized). Plateaus are important to examine in epidemiological studies, as they may provide information on continuing common sources of risk (30), as well as heterogeneity in behavioral and decision-making processes in the populations (31–34).

## Methods

### Data Source

Provisional overdose monthly death counts from the U.S. Centers for Diseases Control and Prevention (CDC) were examined, for the 12-month periods ending June 2019 to November 2021 (therefore, covering a period before and after the onset of COVID-19 in the United States). The selected starting month (June 2019) provided 8 months of pre-COVID-19 data, to characterize a potential minimum plateau, important for non-linear sigmoidal regression analyses. The selected ending month was the latest available at the time of download (20 April 2022; Vital Statistics Rapid Release).^1^ We examined data for the authorities that report overdose deaths caused by “synthetic opioid excluding methadone” (category T40.4) (23). This category contains primarily fentanyl and its analogs, and can potentially include MOR-agonists from other synthetic scaffolds (e.g., analogs of etonitazene and U47700), although the latter are thought to be less common (16, 24). All data from an authority was excluded from non-linear regressions if it had more than two consecutive missing monthly values, or if values for the baseline month (June 2019) were not available.

Data for each 12-month ending period were first plotted per authority over the study period, expressed as crude mortality rate/million of population (to the nearest 0.1 million, based on U.S. Census numbers). Data were not cumulated across authorities, due to the possibility of differences in reporting standards (35, 36). For better visualization, separate figures were plotted for the four Census regions (i.e., West, Midwest, South, and Northeast).

Absolute baseline values (June 2019) differed widely across authorities, and the remaining calculations were made after normalizing each data set’s monthly data as a percent of its baseline month. For each data set, a maximum plateau was defined to occur if *each* of values for the last 4 months (i.e., August 2021–November 2021) was within ±5% of: (mean value for the last 4 months/baseline month). This therefore takes into account the differing magnitudes of increases relative to each authority’s baseline.

### Sigmoidal Time/Mortality Curve

Sigmoidal equations (including the sigmodal Boltzmann model) have been used for epidemiological and opioid clinical pharmacology studies (32, 37, 38). The sigmoidal Boltzmann model was used to fit minimum and maximum (i.e., bottom and top) plateaus for mortality due to synthetic opioids, and the midpoint of the rise in mortality (23, 25). Data sets from the 29 authorities which had a detected maximum plateau in the last 4 months of the study period (as defined above; Table 1) were entered in an overall Boltzmann sigmoidal equation model, using GraphPad Prism (V.9) software. The equation was: mortality = [lower plateau + (top plateau - bottom plateau)]/1 + ^exp^ [(month of midpoint of top - bottom) - month]/slope].^2^ The regression was weighted by 1/Y^2^, to account for larger variation in the later months of the study period. The minimum plateau was constrained to be >0%. The main fitted values of interest were therefore the maximum plateau (±95%CI) and the month of the midpoint between the minimum and maximum plateau (i.e., to determine when the rise in mortality occurred). The minimum plateau is not reported here, as the values were normalized as percent of baseline. This overall sigmoidal model was followed by separate models for the states in the four Census regions, with identical methods.

**TABLE 1:** Mortality rate for overdoses caused by synthetic opioids, before and after the onset of COVID-19.

Census region	Crude mortality rate for synthetic opioids excluding methadone (category T40.4 in CDC Data)					
	
	Authority	Death rate/Million		Ending month as % of baseline month	Mean of last 4 months as % of baseline month	Maximum plateau detected in the last 4 months of study period?^a^
		
		Baseline month: June 2019	Ending month: Nov 2021			
West	California (CA)	29.7	148.4	498.8	492.8	Y
	Alaska (AK)	27.4	204.1	745.0	667.5	**N**
	Arizona (AZ)	85.9	239.3	278.6	275.1	Y
	New Mexico (NM)	55.7	271.0	486.3	466.2	Y
	Colorado (CO)	26.6	163.6	616.2	587.2	**N**
	Hawaii (HI)	12.7	29.3	231.6	211.8	**N**
	Nevada (NV)	34.5	117.7	341.1	330.1	Y
	Oregon (OR)	20.5	103.8	507.0	508.1	Y
	Utah (UT)	25.6	59.4	231.7	220.7	Y
	Wyoming (WY)	16.7	56.7	340.0	325.0	**N**
	Washington (WA)	34.9	150.0	430.2	403.6	**N**
Midwest	Iowa (IA)	23.8	61.3	257.9	269.7	Y
	Illinois (IL)	125.2	209.4	167.2	164.2	Y
	Indiana (IN)	110.1	290.6	263.8	255.6	Y
	Missouri (MO)	137.4	232.6	169.2	165.6	Y
	Ohio (OH)	257.7	357.0	138.5	139.8	Y
	South Dakota (SD)	12.2	37.8	309.1	277.3	**N**
	Wisconsin (WI)	92.9	210.2	226.2	222.4	Y
South	Texas (TX)	10.7	59.0	553.4	529.0	Y
	Maryland (MD)	300.2	372.3	124.0	127.6	Y
	Kansas (KS)	Missing^c^	117.6	N/A	N/A	N/A
	Delaware (DE)	311.0	381.0	122.5	118.2	Y
	Georgia (GA)	31.4	118.9	378.4	360.8	**N**
	Kentucky (KY)	165.8	384.4	231.9	228.3	Y
	Mississippi (MS)	31.7	135.0	426.3	428.2	Y
	North Carolina (NC)	126.4	259.6	205.4	203.3	Y
	Oklahoma (OK)	15.0	64.8	431.7	408.8	**N**
	South Carolina (SC)	98.7	282.3	286.2	279.0	Y
	Tennessee (TN)	147.5	407.2	276.1	267.3	Y
	Virginia (VA)	107.6	220.6	204.9	207.5	Y
	Washington DC (DC)	277.1	532.9	192.3	193.3	Y
	West Virginia (WV)	297.2	637.8	214.6	219.8	Y
Northeast	New York City (NYC)	101.3	222.4^d^	219.5^d^	226.0	N/A
	New York State (NYS)^d^	117.6	196.6	167.2	169.5	Y
	Connecticut (CT)	232.8	359.7	154.5	151.9	Y
	New Jersey (NJ)	237.8	264.3	111.2	111.5	Y
	Massachusetts (MA)	259.6	304.6	117.4	116.3	Y
	Vermont (VT)	145.0	343.3	236.8	225.6	**N**
	Rhode Island (RI)	200.9	286.4	142.5	139.7	Y
	New Hampshire (NH)	260.0	222.1	85.4	86.1	N/A^e^
	Maine (ME)	167.9	347.9	207.2	194.8	**N**

*^a^A maximum plateau is detected if each of the last 4 months of the study period (August 2021–November 2021) are within ± 5% of (mean of last 4 months/baseline month).*

*^b^June–July 2019 data missing.*

*^c^Ending month (November 2021) missing; data shown with October 2021 as ending month.*

*^d^New York State excluding New York City.*

*^e^New Hampshire showed a decrease in mortality rate due to synthetic opioids over this period.*

## Results

**FIGURE 1:**
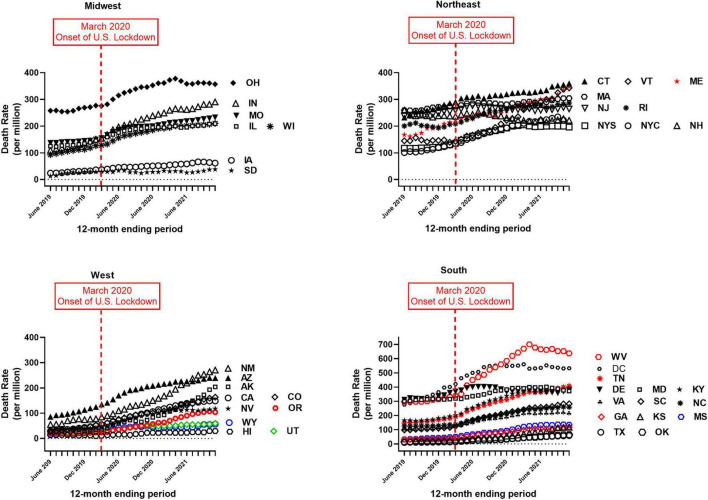
Crude monthly mortality rate (per million), due to “synthetic opioids excluding methadone,” for June 2019–November 2021. *X*-axes: month; *Y*-axes: mortality rate (per million population). Different panels show data separated by Census region (Midwest, Northeast, West and South). Note different Y-axis scale in the panel for the South region (lower right). Labels for each authority are placed near the ending month data for each curve, to aid visualization. Abbreviations for authorities are defined in Table 1.

**FIGURE 2:**
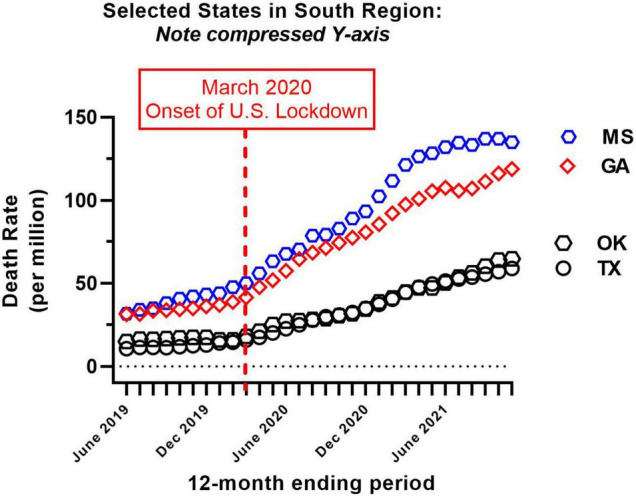
Re-plot of crude mortality rate of the four states from the South census region with the lowest absolute values (i.e., see Figure 1, lower right panel, for comparison). Note compressed Y-axis range; all other details as in Figure 1.

Provisional overdose deaths due to synthetic opioids excluding methadone were analyzed, before and after the onset of COVID-19 in the United States (June 2019–November 2021). Monthly crude mortality rates/million are shown in Figure 1, separated by Census region. There was a considerable variation in baseline mortality rate (Table 1 and Figure 1). Of the 41 authorities studied, only New Hampshire showed a decrease in mortality rate over the study period (to 85% of baseline; Table 1). All other authorities reported an increase in mortality over the study period, and there was a considerable variation in the magnitude of time-dependent increases compared to each baseline (i.e., June 2019; Table 1 and Figure 1). Due to this variation, the potential sigmodal profile of some curves is less easily discernible than others, on a common y-axis axis. As an illustration, the four States in the South census region with lower absolute values are re-plotted on a compressed y-axis (Figure 2, compare to Figure 1, lower right panel).

### Normalized Mortality Rates

Crude mortality rates were then normalized as a percent of each data set’s baseline month (June 2019), and are presented in Table 1. As mentioned above, as of the final month of the study period (Nov 2021), only New Hampshire had a decrease (85% of baseline). All other data sets exhibited increases by the end of the study period, ranging in magnitude from 111 to 645% of baseline (New Jersey and Alaska, respectively).

### Determination of Maximum Plateau in Overdose Mortality

The presence of a maximum plateau (i.e., a period of stability) in the last 4 months of the study period was examined for each data set. There were a total of 39 data sets which could be analyzed (New York City and Kansas were excluded due to missing data in some cells). Of these 39 data sets, 29 showed a maximum plateau as defined in the Methods (Table 1). By contrast, data sets from 10 states did not exhibit a maximum plateau in the last 4 months of the study; these were: Alaska, Colorado, Hawaii, Wyoming, Washington, South Dakota, Georgia, Oklahoma, Vermont, and Maine.

### Sigmoidal Model of Data From States With a Maximum Plateau in Mortality

**FIGURE 3:**
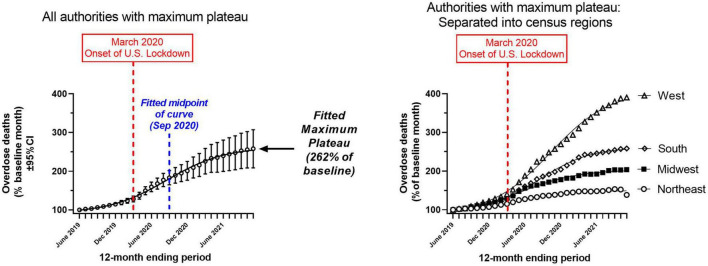
Sigmoidal regressions of mortality, normalized as percent of the baseline month (June 2019) for the 29 authorities which had a maximum plateau in the last 4 months of the study period (August 2021–November 2021; Table 1). Left panel: Overall regression for these 29 authorities. Right panel: Separate regressions for four census regions; confidence intervals not shown. *X*-axes: month; *Y*-axes: mortality rate (percent of baseline month). Table 2 shows the parameters of the sigmoidal fits.

In order to examine the plateau phenomenon further, we entered data from the 29 authorities (28 states and Washington DC) with a detected maximum plateau (see Table 1) into an overall regression, as replicates. For this overall regression, the data fit a sigmoidal Boltzmann equation with a high weighted R^2^ (0.99) (Figure 3, left panel). The fitted maximum plateau for this overall regression was 262% of baseline (95%CI: 255–271%) (Figure 3, left panel). The fitted mid-point between the minimum and maximum plateau was detected in September 2020 (Table 2). In a follow up analysis, we divided these 29 authorities into their four respective Census regions, and carried out separate regressions (Figure 3, right panel; Table 2). These four regressions revealed that the regions had different maximum plateau levels, based on lack of overlap in their weighted 95%CI (Table 2). The region with the highest plateau was the West, followed in descending order by the South, Midwest and Northeast. These region-specific regressions also exhibited high weighted R^2^ (≈0.99).

**TABLE 2:** Non-linear sigmoidal regressions for overdose mortality caused by synthetic opioids.

Sigmoidal non-linear regressions for normalized data (expressed as % of baseline month)^a^		

	**Maximum plateau as % of baseline (weighted ± 95%CI)^b^**	**Midpoint between minimum and maximum plateau^c^**
29 U.S. Authorities combined	262% (255–271)	September 2020
**Separate census regions**		
West region	409% (392–433)	October 2020
South region	262% (256–268)	August 2020
Midwest region	204% (199–209)	May 2020
Northeast region	149% (147–152)	May 2020

*^a^Includes data from 29 authorities which showed a stable maximum plateau in the last 4 months of the study period (described in Table 1).*

*^b^These data describe the maximum (top) plateau as fitted with the sigmoidal Boltzmann regression from June 2019 to November 2021. This does not necessarily imply that these plateaus will be the maximum for the future (see section “Discussion; Limitations and methodological considerations”).*

*^c^To the nearest whole month.*

## Discussion

Several studies have reported an increase in opioid-induced mortality after the onset of COVID-19 in the United States (commencing in March 2020), largely ascribed to synthetic MOR-agonists such as fentanyl analogs (10, 23, 24). Mortality had also been rising in prior years (e.g., a wave in 2014 onwards) due to synthetic MOR-agonists, heroin and prescription opioids (11, 14, 17, 23, 39).

As expected, nearly all of the 41 authorities under study exhibited an increase in mortality due to synthetic opioids after the onset of the COVID-19 lockdowns in March 2020. One authority only (New Hampshire) showed a decrease at the end of the study period. This study detected that 29 of these authorities exhibited a plateau in mortality caused by synthetic opioids, defined as stability in the last 4 months of this period (August 2021–November 2021). The data also show that 10 authorities had not exhibited such a plateau at the end of the study period.

A large variability in baseline mortality due to synthetic opioids and the magnitude of the post-COVID-19 increases was noted across authorities and regions. Overall, the highest plateau was observed in the West Census region, followed in descending order by South, Midwest and Northeast. Variability in these profiles is consistent with recent reports focusing on all opioid overdoses (i.e., not only those due to synthetic opioids), for selected states (25, 26, 40). Future studies could examine the relationship between different relative increases in mortality across regions and States, and diverse public health measures enacted after the onset of COVID-19. Pre-existing differences across regions and States could also be important factors, based on variations in health systems, socioeconomics, demographics and frequency of illicit synthetic opioids in the drug supply (41–46).

Using the pre-pandemic period from June 2019 as baseline, the sigmoidal regression models in this study document an increase in mortality due to synthetic opioids in the United States after the onset of COVID-19 (in March 2020 onwards). For the overall regression, the midpoint of this increase was detected in September 2020, approximately 6 months after the onset of the lockdowns in the United States. The increase of such overdoses during the early pandemic may have been due to changes in supply, distribution or demand, for illicit opioids and other drugs (41, 47). Increased adulteration with fentanyl analogs in different types of illicit drug products (including both opioid and non-opioid compounds) is another potential cause of this trend (39, 45, 48). Behavioral changes in the early pandemic period, such as injecting alone, or refusal of transport to the ED may have also contributed to this rise in mortality (5, 49).

The presence of plateaus in epidemiological studies has been ascribed to different phenomena, including the presence of a “continuing common source,” which in this case could be the extensive penetration of synthetic opioids in the illicit drug supply (30, 39). Other factors that have been recently examined in such plateaus include behaviorally based heterogeneity within populations, and behavioral change due to awareness of risk (31, 34).

### Limitations and Methodological Considerations

It cannot be excluded that the plateaus observed at the end of the study period in 29 authorities are temporary, and that further increases may occur in the future (17, 31, 34, 50). Only one state examined here, New Hampshire, showed a decrease in mortality due to synthetic opioids, compared to baseline. A slight time-dependent decrease was also observed for New Hampshire for mortality due to all opioids (T40.0–T40.4, T40.6), not only synthetic opioids studied here (not shown).

The analysis for “synthetic opioids excluding methadone” (category T40.4) did not include all States, as some do not report this category separately. Nevertheless, this study included a large sample of reporting authorities, representing all census regions in the United States. These CDC mortality data are considered provisional, and have lag times for reporting (51); there are also potential differences in reporting standards across authorities (36), further justifying normalization by the local baseline. However, completeness is relatively high, and these data are among the most timely indicators of overdose mortality across the United States (see text footnote 1).

The available data do not allow differentiation of which specific synthetic opioid (e.g., fentanyl or an analog thereof) was detected (24). Also, these data sets do not imply that “synthetic opioids other than methadone” were the only drugs detected in the decedents (52). However synthetic opioids are thought to be the compounds primarily underlying the increases in mortality observed after the onset of COVID-19 (14, 41). For illustration, California (the most populous State) had a *decrease* in overdose mortality with heroin (T40.1) in the last month of the study period compared to baseline (to 80.9% of baseline; not shown). By contrast, there had been a robust increase in overdose mortality with “synthetic opioids other than methadone” (i.e., 498.8% of baseline; Table 1).

Because there was a broad variation across authorities in the crude mortality rate at baseline (i.e., June 2019), data were normalized data as percent of each baseline, for sigmoidal non-linear regressions. However a similar sigmoidal curve could also be fit on crude mortality rates (not shown), therefore the models shown here were not simply an artifact of normalization. Modeling of these monthly data with a sigmoidal Boltzmann equation was justified based on the observed profiles in most of the authorities in this study, and has been used to examine other epidemiological variables (32, 37). Nevertheless, equations other than the sigmoidal Boltzmann could be used in the future to explore this potential plateau phenomenon. In the sigmoidal models shown here, we included only authorities that had individually shown a stable maximum plateau in the last 4 months of the study period. As a follow-up, we also carried out a further Boltzmann model including all data sets without exclusion. Results of this equation were also fit to a sigmoidal model with high R^2^, and yielded similar maximum plateau to that reported above (not shown). Therefore, the fitted sigmoidal models here were not likely to be an artifact of the exclusion of the data sets that had not individually reached a plateau.

## Conclusion

The COVID-19 pandemic has potentially affected different stages in OUD, including initiation, escalation, overdoses, and engagement with clinical care (3, 5, 21). We confirm here increases in mortality rates due to synthetic opioids after the onset of COVID-19 in the United States, which varied considerably in magnitude across census regions and the broad array of states examined here. This study shows that most, but not all, of the authorities had reached a stable plateau in August to November 2021. The magnitude of the observed plateau was the highest for the West census region, followed in descending order by South, Midwest and Northeast. Differing plateau levels in specific parts of the United States may be due to patterns of opioid initiation and use, but also use of other drugs, as illicit synthetic opioids can be mixed in the drug supply for ostensibly non-opioid drugs (14, 39, 53). The lack of observable plateaus at the end of the study period in 10 of the states studied here is also worthy of investigation, given the potential risk of further increases. Overall, future studies should explore the reasons underlying these divergent plateau profiles across states, as a potential guide for locally optimized prevention, mitigation and intervention approaches.

## Data Availability Statement

Publicly available datasets were analyzed in this study. This data can be found here: https://www.cdc.gov/nchs/nvss/vsrr/drug-overdose-data.htm.

## Author Contributions

All authors listed have made a substantial, direct, and intellectual contribution to the work, and approved it for publication.

## Conflict of Interest

The authors declare that the research was conducted in the absence of any commercial or financial relationships that could be construed as a potential conflict of interest.

## Publisher’s Note

All claims expressed in this article are solely those of the authors and do not necessarily represent those of their affiliated organizations, or those of the publisher, the editors and the reviewers. Any product that may be evaluated in this article, or claim that may be made by its manufacturer, is not guaranteed or endorsed by the publisher.

## Footnotes

1
https://www.cdc.gov/nchs/nvss/vsrr/drug-overdose-data.htm

2
https://www.graphpad.com/guides/prism/latest/curve-fitting/REG_Classic_Boltzmann.htm

## Abbreviations

95%CI: 95% confidence interval
COVID-19: Coronavirus disease 2019
CDC: U.S. Centers for Disease Control and Preventions
ED: Emergency Department
MOR: mu-opioid receptor
OUD: opioid use disorder.

## References

[1] Kates J Michaud J Tolbert J. *Stay-At-Home Orders to Fight COVID-19 in the United States: The Risks of a Scattershot Approach.* (2020). Available online at: https://www.kff.org/policy-watch/stay-at-home-orders-to-fight-covid19/ (accessed June 7, 2021).

[2] Wang QQ Kaelber DC Xu R Volkow ND. COVID-19 risk and outcomes in patients with substance use disorders: analyses from electronic health records in the United States. *Mol Psychiatry.* (2020) 26:40. 10.1038/s41380-020-00880-7 32929211 PMC7488216

[3] Bart G Wastvedt S Hodges JS Rosenthal R. Did drug use increase following COVID-19 relaxation of methadone take-out regulations? 2020 was a complicated year. *J Subst Abuse Treat.* (2022) 133:108590. 10.1016/j.jsat.2021.108590 34373169 PMC8343384

[4] Nunes EV Levin FR Reilly MP El-Bassel N. Medication treatment for opioid use disorder in the age of COVID-19: can new regulations modify the opioid cascade? *J Subst Abuse Treat.* (2021) 122:108196. 10.1016/j.jsat.2020.108196 33221125 PMC7666540

[5] Root ED Slavova S LaRochelle M Feaster DJ Villani J Defiore-Hyrmer J The impact of the national stay-at-home order on emergency department visits for suspected opioid overdose during the first wave of the COVID-19 pandemic. *Drug Alcohol Depend.* (2021) 228:108977. 10.1016/j.drugalcdep.2021.108977 34598100 PMC8397502

[6] Ding H Trapella C Kiguchi N Hsu FC Caló G Ko MC. Functional profile of systemic and intrathecal cebranopadol in nonhuman primates. *Anesthesiology.* (2021) 135:482–93. 10.1097/aln.0000000000003848 34237134 PMC8446297

[7] France CP Ahern G Averick S Disney A Enright HA Esmaeli-Azad B Countermeasures for preventing and treating opioid overdose. *Clin Pharmacol Ther.* (2020) 109:578–90. 10.1002/cpt.2098 33113208 PMC8193687

[8] Liu S Kim DI Oh TG Pao GM Kim JH Palmiter RD Neural basis of opioid-induced respiratory depression and its rescue. *Proc Natl Acad Sci USA.* (2021) 118:e2022134118. 10.1073/pnas.2022134118 34074761 PMC8201770

[9] Yang PK Weinger MB Negus SS. Elucidation of dose-effect relationships for different opiate effects using alfentanil in the spontaneously ventilating rat. *Anesthesiology.* (1992) 77:153–61. 10.1097/00000542-199207000-00022 1609989

[10] Britch SC Walsh SL. Treatment of opioid overdose: current approaches and recent advances. *Psychopharmacology (Berl).* (2022) 239:2063–81. 10.1007/s00213-022-06125-5 35385972 PMC8986509

[11] Comer SD Cahill CM. Fentanyl: receptor pharmacology, abuse potential, and implications for treatment. *Neurosci Biobehav Rev.* (2019) 106:49–57. 10.1016/j.neubiorev.2018.12.005 30528374 PMC7233332

[12] Lovrecic B Lovrecic M Gabrovec B Carli M Pacini M Maremmani AGI Non-medical use of novel synthetic opioids: a new challenge to public health. *Int J Environ Res Public Health.* (2019) 16:177. 10.3390/ijerph16020177 30634521 PMC6352208

[13] Schifano F Chiappini S Corkery JM Guirguis A. Assessing the 2004-2018 fentanyl misusing issues reported to an international range of adverse reporting systems. *Front Pharmacol.* (2019) 10:46. 10.3389/fphar.2019.00046 30774595 PMC6367955

[14] CDC. *Increase in Fatal Drug Overdoses Across the United States Driven by Synthetic Opioids Before and During the COVID-19 Pandemic.* Atlanta, GA: CDC (2020).

[15] Rudd RA Aleshire N Zibbell JE Gladden RA. Increases in drug and opioid overdose deaths — United States, 2000–2014. *Morb Mortal Wkly Rep.* (2015) 64:1378–82. 10.15585/mmwr.mm6450a3 26720857

[16] Seth P Rudd RA Noonan RK Haegerich TM. Quantifying the epidemic of prescription opioid overdose deaths. *Am J Public Health.* (2018) 108:500–2. 10.2105/ajph.2017.304265 29513577 PMC5844400

[17] Ciccarone D. The triple wave epidemic: supply and demand drivers of the US opioid overdose crisis. *Int J Drug Policy.* (2019) 71:183–8. 10.1016/j.drugpo.2019.01.010 30718120 PMC6675668

[18] Cicero TJ Ellis MS Kasper ZA. Increases in self-reported fentanyl use among a population entering drug treatment: the need for systematic surveillance of illicitly manufactured opioids. *Drug Alcohol Depend.* (2017) 177:101–3. 10.1016/j.drugalcdep.2017.04.004 28582697

[19] Henry BF Mandavia AD Paschen-Wolff MM Hunt T Humensky JL Wu E COVID-19, mental health, and opioid use disorder: old and new public health crises intertwine. *Psychol Trauma.* (2020) 12:S111–2. 10.1037/tra0000660 32551759 PMC7583654

[20] Kosten TR Petrakis IL. The hidden epidemic of opioid overdoses during the coronavirus disease 2019 pandemic. *JAMA Psychiatry.* (2021) 78:585–6. 10.1001/jamapsychiatry.2020.4148 33377967

[21] Volkow ND. Collision of the COVID-19 and addiction epidemics. *Ann Intern Med.* (2020) 173:61–2. 10.7326/m20-1212 32240293 PMC7138334

[22] Wakeman SE Green TC Rich J. An overdose surge will compound the COVID-19 pandemic if urgent action is not taken. *Nat Med.* (2020) 26:819–20. 10.1038/s41591-020-0898-0 32555514 PMC8600654

[23] Ahmad FB Rossen LM Sutton P. *Provisional Drug Overdose Death Counts.* Hyattsville, MD: National Center for Health Statistics (2021).

[24] Skolnick P. Treatment of overdose in the synthetic opioid era. *Pharmacol Ther.* (2021) 233:108019. 10.1016/j.pharmthera.2021.108019 34637841

[25] Garcia GP Stringfellow EJ DiGennaro C Poellinger N Wood J Wakeman S Opioid overdose decedent characteristics during COVID-19. *Ann Med.* (2022) 54:1081–8. 10.1080/07853890.2022.2067350 35467475 PMC9045762

[26] Burgess-Hull AJ Smith KE Panlilio LV Schriefer D Preston KL Alter A Nonfatal opioid overdoses before and after Covid-19: regional variation in rates of change. *PLoS One.* (2022) 17:e0263893. 10.1371/journal.pone.0263893 35263326 PMC8906602

[27] Larson PS Bergmans RS. Impact of the COVID-19 pandemic on temporal patterns of mental health and substance abuse related mortality in Michigan: an interrupted time series analysis. *Lancet Reg Health Am.* (2022) 10:100218. 10.1016/j.lana.2022.100218 35284903 PMC8898171

[28] Macmadu A Batthala S Correia Gabel AM Rosenberg M Ganguly R Yedinak JL Comparison of characteristics of deaths from drug overdose before vs during the COVID-19 pandemic in Rhode Island. *JAMA Netw Open.* (2021) 4:e2125538. 10.1001/jamanetworkopen.2021.25538 34533569 PMC8449276

[29] Shreffler J Shoff H Thomas JJ Huecker M. Brief report: the impact of COVID-19 on emergency department overdose diagnoses and county overdose deaths. *Am J Addict.* (2021) 30:330–3. 10.1111/ajad.13148 33738889 PMC8250732

[30] Fontaine RE. *Describing Epidemiologic Data.* Atlanta, GA: CDC (2019).

[31] Berestycki H Desjardins B Heintz B Oury J-M. Plateaus, rebounds and the effects of individual behaviours in epidemics. *Sci Rep.* (2021) 11:18339. 10.1038/s41598-021-97077-x 34526528 PMC8443568

[32] El Aferni A Guettari M Tajouri T. Mathematical model of Boltzmann’s sigmoidal equation applicable to the spreading of the coronavirus (Covid-19) waves. *Environ Sci Pollut Res Int.* (2021) 28:40400–8. 10.1007/s11356-020-11188-y 33058082 PMC7557153

[33] Smaldino PE Aplin LM Farine DR. Sigmoidal acquisition curves are good indicators of conformist transmission. *Sci Rep.* (2018) 8:14015. 10.1038/s41598-018-30248-5 30228351 PMC6143626

[34] Weitz JS Park SW Eksin C Dushoff J. Awareness-driven behavior changes can shift the shape of epidemics away from peaks and toward plateaus, shoulders, and oscillations. *Proc Natl Acad Sci USA.* (2020) 117:32764–71. 10.1073/pnas.2009911117 33262277 PMC7768772

[35] Slavova S O’Brien DB Creppage K Dao D Fondario A Haile E Drug overdose deaths: let’s get specific. *Public Health Rep.* (2015) 130:339–42. 10.1177/003335491513000411 26345488 PMC4547584

[36] Warner M Paulozzi LJ Nolte KB Davis GG Nelson LS. State variation in certifying manner of death and drugs involved in drug intoxication deaths. *Acad Forensic Pathol.* (2013) 3:231–7. 10.23907/2013.029

[37] Hartwig TS Sørensen S Jørgensen FS. The maternal age-related first trimester risks for trisomy 21, 18 and 13 based on Danish first trimester data from 2005 to 2014. *Prenatal Diagnosis.* (2016) 36:643–9. 10.1002/pd.4833 27135649

[38] Olofsen E Boom M Sarton E van Velzen M Baily P Smith KJ Analgesic and respiratory depressant effects of R-dihydroetorphine: a pharmacokinetic-pharmacodynamic analysis in healthy male volunteers. *Anesthesiology.* (2019) 131:1327–39. 10.1097/aln.0000000000002991 31651529

[39] Fairbairn N Coffin PO Walley AY. Naloxone for heroin, prescription opioid, and illicitly made fentanyl overdoses: challenges and innovations responding to a dynamic epidemic. *Int J Drug Policy.* (2017) 46:172–9. 10.1016/j.drugpo.2017.06.005 28687187 PMC5783633

[40] Holland KM Jones C Vivolo-Kantor AM Idaikkadar N Zwald M Hoots B Trends in US emergency department visits for mental health, overdose, and violence outcomes before and during the COVID-19 pandemic. *JAMA Psychiatry.* (2021) 78:372–9. 10.1001/jamapsychiatry.2020.4402 33533876 PMC7859873

[41] Ciccarone D. The rise of illicit fentanyls, stimulants and the fourth wave of the opioid overdose crisis. *Curr Opin Psychiatry.* (2021) 34:344–50. 10.1097/yco.0000000000000717 33965972 PMC8154745

[42] Friedman J Hansen H Bluthenthal RN Harawa N Jordan A Beletsky L. Growing racial/ethnic disparities in overdose mortality before and during the COVID-19 pandemic in California. *Prev Med.* (2021) 153:106845–106845. 10.1016/j.ypmed.2021.106845 34653501 PMC8521065

[43] Friedman JR Hansen H. Evaluation of Increases in drug overdose mortality rates in the us by race and ethnicity before and during the COVID-19 pandemic. *JAMA Psychiatry.* (2022) 79:379–81. 10.1001/jamapsychiatry.2022.0004 35234815 PMC8892360

[44] Jalal H Buchanich JM Sinclair DR Roberts MS Burke DS. Age and generational patterns of overdose death risk from opioids and other drugs. *Nat Med.* (2020) 26:699–704. 10.1038/s41591-020-0855-y 32367060 PMC8086189

[45] Kilmer B Pardo B Pujol TA Caulkins JP. Rapid changes in illegally manufactured fentanyl products and prices in the United States. *Addiction.* (2022). 10.1111/add.15942 35543081 PMC9543283

[46] Shover CL Falasinnu TO Dwyer CL Santos NB Cunningham NJ Freedman RB Steep increases in fentanyl-related mortality west of the Mississippi River: recent evidence from county and state surveillance. *Drug Alcohol Depend.* (2020) 216:108314. 10.1016/j.drugalcdep.2020.108314 33038637 PMC7521591

[47] Mars SG Rosenblum D Ciccarone D. Illicit fentanyls in the opioid street market: desired or imposed? *Addiction.* (2018) 114:774–80. 10.1111/add.14474 30512204 PMC6548693

[48] Macmadu A Carroll JJ Hadland SE Green TC Marshall BD. Prevalence and correlates of fentanyl-contaminated heroin exposure among young adults who use prescription opioids non-medically. *Addict Behav.* (2017) 68:35–8. 10.1016/j.addbeh.2017.01.014 28088741 PMC5291510

[49] Slavova S Rock P Bush HM Quesinberry D Walsh SL. Signal of increased opioid overdose during COVID-19 from emergency medical services data. *Drug and Alcohol Depend.* (2020) 214:108176. 10.1016/j.drugalcdep.2020.108176 32717504 PMC7351024

[50] Jalal H Burke DS. Carfentanil and the rise and fall of overdose deaths in the United States. *Addiction.* (2021) 116:1593–9. 10.1111/add.15260 32935381 PMC8019064

[51] Spencer MR Ahmad F. *Timeliness of Death Certificate Data for Mortality Surveillance and Provisional Estimates.* Washington, DC: National Center for Health Statistics (2016).

[52] CDC. *Provisional Drug Overdose Death Counts.* Atlanta, GA: CDC (2020).

[53] Bolinski RS Walters S Salisbury-Afshar E Ouellet LJ Jenkins WD Almirol E The Impact of the COVID-19 pandemic on drug use behaviors, fentanyl exposure, and harm reduction service support among people who use drugs in rural settings. *Int J Environ Res Public Health.* (2022) 19:2230. 10.3390/ijerph19042230 35206421 PMC8872091

